# CAPHEINE, or Everything and the Kitchen Sink: A Workflow for Automating Selection Analyses Using HyPhy

**DOI:** 10.1093/gbe/evag173

**Published:** 2026-07-17

**Authors:** Hannah Verdonk, Danielle Callan, Sergei Pond

**Affiliations:** Institute for Genomics and Evolutionary Medicine, Temple University, Philadelphia, PA 19122, USA; Institute for Genomics and Evolutionary Medicine, Temple University, Philadelphia, PA 19122, USA; Institute for Genomics and Evolutionary Medicine, Temple University, Philadelphia, PA 19122, USA

**Keywords:** bioinformatics workflow, Nextflow pipeline, HyPhy, viral evolution, molecular evolution, pathogen genomics

## Abstract

Here, we present CAPHEINE, a computational workflow that starts with a set of unaligned pathogen sequences and a reference genome and performs a comprehensive exploratory evolutionary analysis of the input data. CAPHEINE pairs nicely with studies of site-level selection dynamics, gene-level positive selection, and lineage-specific shifts in selective pressure. Our workflow is portable across Mac OS, Windows, and Linux, allowing researchers to focus on results. CAPHEINE is freely available at https://github.com/veg/CAPHEINE, along with a set of usage instructions.

## Introduction

Evolutionary dynamics and transmission mechanisms in viruses are incredibly diverse. This results in significant variability in their patterns of emergence, spread, and persistence (Pybus and Rambaut 2009; Leventhal et al. 2015). RNA viruses, such as Influenza A virus (IAV), Hepatitis C virus (HCV), Human Immunodeficiency Virus Type 1 (HIV-1), and SARS-CoV-2 are all characterized by rapid mutation rates and within-host evolution, allowing them to generate diverse viral variants within a host and generating strains with enhanced immune evasion or transmission potential (Luciani and Alizon 2009; Dingens et al. 2019; Lee et al. 2019). IAV, SARS-CoV-2, and HIV-1 are able to create further genetic diversity through reassortment or homologous recombination (Burke 1997; Jetzt et al. 2000; Steel and Lowen 2014).

Lack of genetic diversity can also play a role in viral evolution and dynamics. New infections are often characterized by transmission bottlenecks, as there is less diversity in the founder virus population than in the original host. The bottleneck can lead to genetic drift and the rise of potentially deleterious variants by chance alone (Markov et al. 2023). Additionally, vector-borne RNA viruses face stronger selective constraints due to their life cycle—dengue virus, for example, must persist in both vertebrate and invertebrate hosts (Holmes 2010; Nanaware et al. 2021).

Understanding how viruses spread and persist requires insight into their evolutionary dynamics. With vast amounts of viral sequence data available from sources such as GISAID and the Sequence Read Archive (SRA) (Shu and McCauley 2017; Katz et al. 2022), there is a pressing need for computational methods that can effectively probe this wealth of information and extract meaningful signals of selection. Such methods must be rapid, flexible, and scalable to accommodate frequent updates with newly available genomic data. A computational workflow is an ideal tool to analyze these data. Well-designed workflows must be documented, reproducible analyses; they are portable across different hardware platforms and operating systems, allowing researchers to focus on the high-level analysis logic and results without worrying about the specific software implementation, version control, or dependencies. Support for HPC schedulers and containerization platforms (eg Docker and Singularity) add flexibility - different analysis steps can be parallelized and isolated from the native OS environment.

Existing computational workflows to characterize viral evolution are often tailored to a particular viral pathogen or dataset, eg (Hadfield et al. 2018; Lucaci et al. 2022), and can be difficult to modify without technical experience. Other, more generalist workflows such as V-pipe and ViralFlow v1.0 (Posada-Céspedes et al. 2021; da Silva et al. 2024) characterize positive selection on single nucleotide variants of interest within a single sample or population, but do not test for (and consequently, may not capture) selection across lineages or at conserved sites.

Here, we introduce the *C*omprehensive *A*utomated *P*ipeline using *H*yPhy for *E*volutionary *I*nference with *Ne*xtflow (CAPHEINE), a computational workflow that characterizes selection across sites, branches, and clades within a set of pathogen sequences.

CAPHEINE is designed for exploratory hypothesis generation rather than definitive inference of adaptive mutations. Our workflow enables researchers to quickly profile sequence variation and establish correlations between selective pressure and amino acid composition. As a demonstration, we apply CAPHEINE to H5N1 IAV genomes isolated between 1975 and 2025 and identify signatures of selection across all viral genes. We also contrast selection across sites within the reservoir population of wild birds (Anseriformes and Charadriiformes) and within recent cattle spillover sequences from the 2025 outbreak, searching for signatures of viral adaptation to cattle hosts.

## Methods

We developed CAPHEINE with the goal of quickly and easily surveying the selection profile for a set of viral genes. Characterizing selection across the coding genome is a central analysis in evolutionary biology, and frequently occurs in studies that characterize genomic evolutionary dynamics, profile the strength and direction (positive or negative) of natural selection, connect evidence of coding sequence selective constraints with protein structure and function, and explore how shifts in selection pressure correlate with host changes (Langedijk et al. 2024; Siozios et al. 2024; Balech et al. 2025; Kotwa et al. 2025). In a recent paper in *PLOS Pathogens*, Huang *et al.* characterize the antiviral mechanism of Zinc finger antiviral protein (ZAP) (Huang et al. 2024). The authors identify candidate viral interaction sites in mammalian ZAP orthologs by identifying sites with statistical evidence of positive selection. Subsequent *in vitro* mutagenesis at each candidate site revealed one mutant (N658A) with enhanced antiviral activity over the wild-type ZAP protein, confirming the functional importance of one candidate.

**Fig. 1.**
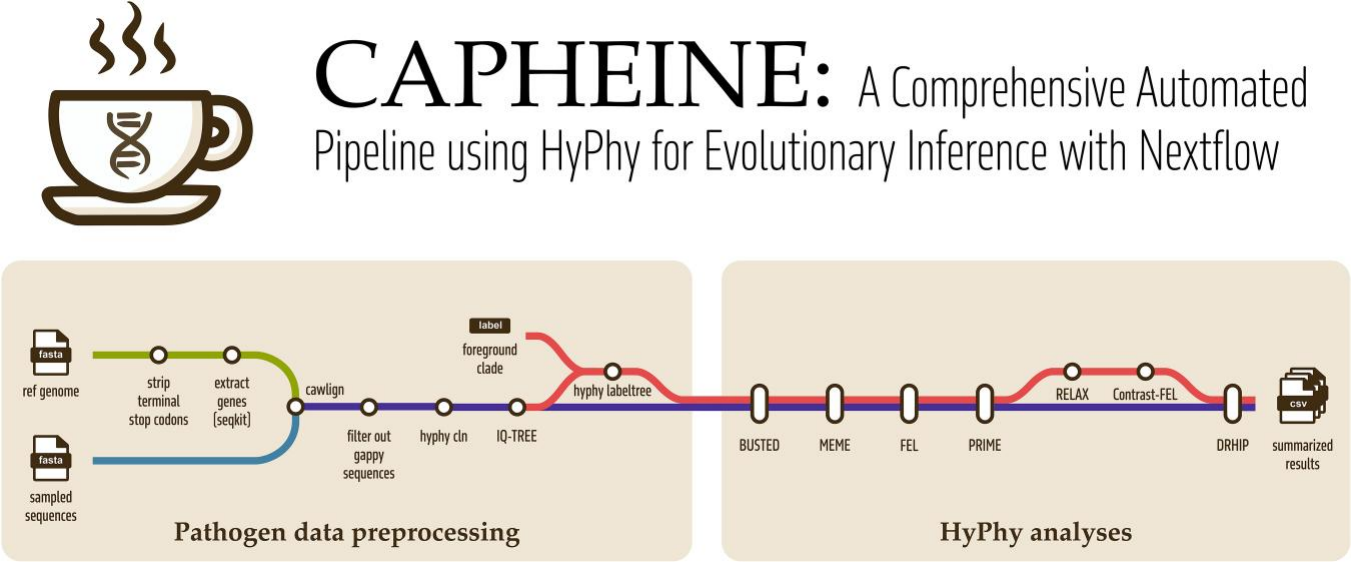
Overview of the CAPHEINE workflow, including data preprocessing steps and evolutionary analyses. There are two sources of input data: a reference genome and the pathogen sequences to be analyzed. Once the sequences are aligned to the reference genome (cawlign), the data undergoes further preprocessing and analysis. Optionally, a set of foreground taxa may also be provided, triggering the additional evolutionary analyses Contrast-FEL and RELAX.

CAPHEINE streamlines the evolutionary discovery process by providing a single, standardized workflow that performs selection analyses quickly, reproducibly, and at scale (Fig. 1). While the underlying statistical models (such as BUSTED, MEME, and FEL) are well-established, their standalone execution requires a complex, multi-step sequence of sequence preprocessing, alignment, tree construction, name sanitization, and manual file format conversion. CAPHEINE standardizes and automates these steps into a single, cohesive workflow. By wrapping these methods under the strict reproducibility and containerization standards of the nf-core framework, CAPHEINE lowers the barrier to entry for evolutionary analysis and eliminates the use of ad-hoc, lab-specific scripting which frequently compromises reproducibility. Many of the studies cited here have independently invested substantial effort in constructing custom pipelines for similar analyses, creating separate workflows that might be difficult to reproduce or compare across studies. CAPHEINE ensures consistency and transparency throughout all analyses by basing itself on the nf-core framework, a standardized set of tools and guidelines for writing bioinformatics pipelines in the Nextflow workflow language (Di Tommaso et al. 2017; Ewels et al. 2019). The CAPHEINE workflow takes just two input files: a “query” dataset comprising a single FASTA file containing unaligned full or partial pathogen genomes, and a “reference” dataset of gene sequences for the pathogen in fasta format (eg downloaded genomic coding sequences from NCBI RefSeq assembly GCF_000864105.1). As a result, users can begin running CAPHEINE with little or no additional data preparation.

Optionally, users may also specify a subset of *foreground* sequences to test for differences in selection pressure against all other sequences. Contrasting the evolutionary history between two branch sets allows users to explore how selection intensity changes with lineage, as well as how individual sites evolve differently under different circumstances. The work of Kotwa *et al.* provides a case study: the authors contrast selection in the S (spike) gene for a newly discovered *Eptesicus fuscus-*derived bat coronavirus and closely related sequences, and for a newly discovered *Myotis lucifugus-*derived bat coronavirus and closely related sequences (Kotwa et al. 2025). Using a combination of HyPhy methods, Kotwa *et al.* find that selection acts in a broadly similar manner for both the newly discovered coronaviruses and their most closely related sequences; there is no evidence for relaxed or intensified selection, and only one site in the *Eptesicus fuscus-*derived bat clade showed statistically significant evidence differential selection between the newly discovered coronavirus and its relatives.

To perform a similar analysis, taxa (or branches) can be marked as foreground lineages with either a list of newline-separated sequence identifiers, or with a regular expression pattern that can be used to match the foreground sequence identifiers. CAPHEINE will report any relaxation or intensification of selection between foreground and reference lineages, as well as site-specific differences in selection pressure and amino acid composition between foreground and reference. The foreground taxa need not be monophyletic or consistently present across all genes.

CAPHEINE first trims the terminal stop codons (if needed) from the reference sequences and removes query sequences with more than 50% gaps or ambiguous bases (Fig. 1). Then, query sequences are mapped to each reference gene using cawlign v0.1.14 (-s BLOSUM62 -t codon). Cawlign is a codon-aware pairwise alignment algorithm based on dynamic programming with frame-preserving gap penalties and explicit modeling of codon triplets, ensuring reading frame integrity during alignment (Anon 2025). Duplicate sequences are removed from each gene’s alignment using hyphy cln, which speeds up computation without affecting any hyphy methods’ estimate of selection. A maximum likelihood tree is then inferred for each alignment using IQ-Tree2 v2.4.0 (-T 8 -m GTR+I+G) (Minh et al. 2020). Although model selection (eg ModelFinder) and branch support estimation (such as bootstrapping) could be performed, we prioritize computational throughput for large pathogen datasets and thus estimate a single maximum-likelihood tree under the GTR+I+G model. Because HyPhy subsequently re-estimates all branch lengths under a codon-model framework, the selection analyses are generally robust to minor nucleotide model misspecification. In practice, standard selection tests (such as BUSTED, FEL, and MEME) are robust to minor topological errors, although major node rearrangements can bias rate estimates. Similarly, contrastive tests (Contrast-FEL and RELAX) are generally robust to topological uncertainty at the tips of the tree, provided that the overall branch set assignments are stable; however, these tests can be sensitive to topological errors if the foreground or reference branch sets are small. We recommend that users with highly uncertain phylogenies verify their results using alternative topologies. If a foreground clade is provided, internal branches of the foreground clade are labeled as “Foreground” and internal branches of the background clade are labeled as “Reference” using the label-tree function in HyPhy v2.5.84 (--internal-nodes ‘‘All descendants’’ --leaf-nodes ‘‘Skip’’) (Kosakovsky Pond et al. 2020). Optionally, users can label all branches (including leaf branches) belonging to the foreground and reference clades, respectively.

We have selected six molecular evolutionary analyses (Spielman et al. 2019), implemented in the HyPhy package, as the core of our workflow (Table 1). These analyses were chosen because they are popular and frequently used in other studies (West et al. 2021; Huang et al. 2024; Policarpo et al. 2024; Balech et al. 2025; Drabeck et al. 2025). Note that Contrast-FEL and RELAX are only run if a foreground set of branches is provided. Additionally, site-specific selection rates (such as *ω*) typically exhibit high variance and boundary constraints under finite sequence alignments, making direct comparison of point estimates or their confidence intervals numerically unstable. Consequently, CAPHEINE relies on Likelihood Ratio Testing (LRT) frameworks—such as Contrast-FEL—which statistically compare selection pressures by evaluating nested models rather than relying on point-estimate comparisons. The statistical properties, Type I error control, and power of these integrated HyPhy methods have been extensively characterized in their respective publications. Fixed Effects Likelihood (FEL) has been shown to strictly control the Type I error rate under neutral simulations (with empirical false positive rates [FPR] at or below the nominal *α* level) and maintains robust power for pervasive selection as sequence counts grow (Kosakovsky Pond and Frost 2005). The Mixed Effects Model of Evolution (MEME) maintains similar nominal Type I error control (≤5% at α=0.05) but provides substantially higher statistical power (often exceeding 80%) to identify transient, episodic selection occurring on only a subset of lineages (Murrell et al. 2012). At the gene level, the Branch-site Unrestricted Statistical Test for Episodic Diversification (BUSTED) is statistically conservative (FPR of 1% TO 3% at α=0.05) under neutral null scenarios while achieving near 100% power to detect episodic diversifying selection under moderate-to-high sequence divergence (Murrell et al. 2015). For detecting relaxed selection, RELAX controls false-positive rates at nominal levels and demonstrates up to 100% power to identify selection relaxation (k≤0.5) or intensification (k≥2.0) across predefined clades (Wertheim et al. 2014). Finally, Contrast-FEL controls the Type I error rate (≤5% at α=0.05) while exhibiting statistical power exceeding 90% to identify differential selection pressures between branch groups when selective differences are sufficiently large, requiring at least 10 branches in each defined set to achieve stable performance (Kosakovsky Pond et al. 2021).

**Table 1.** A summary of all the evolutionary methods included in CAPHEINE

Method	Tests for	Details	Citation
FEL	pervasive (ie phylogeny-wide) diversifying selection at each site in an alignment.	Statistical significance is assessed by LRT and reported at the site level. Benjamini–Hochberg false discovery rate correction is applied at the site level.	(Kosakovsky Pond and Frost 2005)
MEME	episodic diversifying selection—ie diversifying selection that has occurred on only a single branch, or subset of branches in the phylogeny along a site.	Statistical significance is assessed by LRT and reported at the site level. Benjamini–Hochberg false discovery rate correction is applied at the site level.	(Murrell et al. 2012)
BUSTED	gene-wide evidence of positive episodic diversifying selection	Statistical significance is assessed by LRT and reported at the gene level.	(Murrell et al. 2015)
PRIME	whether changes in amino acid properties (eg hydrophobicity) at a site are favored or disfavored	We have chosen to focus on the set of five biochemical properties described by Atchley *et al.* (Atchley et al. 2005) when assessing property conservation at each site.	Forthcoming
Contrast-FEL	Whether a site evolves differently on a specified foreground clade, relative to the set of reference branches	Statistical significance is assessed by LRT and reported at the site level. Benjamini–Hochberg false discovery rate correction is applied at the site level.	(Kosakovsky Pond et al. 2021)
RELAX	whether the strength of selection has been relaxed or intensified gene-wide on a specified foreground clade, relative to the set of reference branches	RELAX is **not** designed to test a set of lineages for the presence or absence of diversifying selection, but to find relaxation (K<1) or intensification (K>1) of selection on the foreground branches relative to the reference branches.	(Wertheim et al. 2014)

Contrast-FEL and RELAX are only run if a foreground clade is provided.

## Case Study: H5N1 Host Shift

Here, we demonstrate how to run CAPHEINE and interpret the output using H5N1 IAV viral sequences collected between 1975 and August 12, 2025 and uploaded to GenBank. Running CAPHEINE with default parameters allows us to explore broad, gene- and site-level patterns of selection across our entire dataset, so that we can see if the patterns we find are consistent with what we already know about IAV evolutionary dynamics. However, we also wish to find sites that might be under differential selection due to the host shift from the wild bird reservoir population to the cattle outbreak population. To that end, we specify cattle isolates as our foreground taxa with the additional argument -foreground_regexp  ‘‘cattle’’; the regular expression will match all fasta IDs that contain the string “cattle” and mark those sequences as foreground. We have chosen to set a significance threshold of q<0.2 to facilitate We have set a less stringent significance threshold of q<0.2 to facilitate exploratory analysis of our dataset and to prioritize detection of weaker signals of selection. CAPHEINE outputs *q*-values for all sites, so users may set more relaxed or more stringent thresholds for their own data.

Our starting dataset included 28,751 genomic sequences from over 2,706 cattle isolates and 41,988 genomic sequences from over 3,396 wild bird (*Anseriformes* and *Charadriiformes*) isolates. We are unsure of the exact number of isolates due to isolate naming inconsistencies in Genbank, so we report the number of isolates with exactly 8 associated segments per dataset. NCBI’s RefSeq datasets provide users with the option of downloading reference coding sequences for annotated genomes; we took advantage of this feature to obtain H5N1 IAV reference gene sequences (NCBI RefSeq assembly GCF_000864105.1). We excluded the short overlapping PA-X and PB1-F2 genes from our analyses. After duplicate removal with hyphy cln, each gene alignment contained between 980 and 4,600 unique sequences (Table S1).

We restricted the HyPhy analyses to include only *internal branches* to capture fixed differences due to selection for transmission and survival, rather than intra-host evolution. Users may configure CAPHEINE to analyze all branches if desired. Analyses also assume the Universal genetic code for all aligned sequences, but this can be easily reconfigured.

CAPHEINE summarizes and reports all analysis results as plain-text CSV files. These files can be readily opened in common spreadsheet programs, allowing users to easily inspect or explore the data without requiring specialized software or advanced computational expertise. Results files also drop directly into downstream analytics in R, Python, or visualization notebooks, enabling rapid iteration from first pass results to figures suitable for reports and manuscripts.

**Fig. 2.**
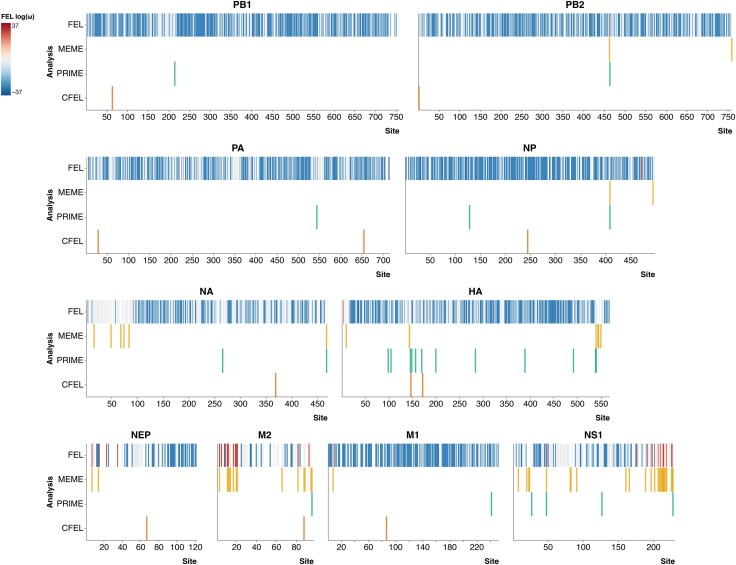
Site-wise CAPHEINE results. Significant sites for each method are shown as colored bars, while insignificant sites are left blank. For FEL, direction of selection (positive or negative) is shown as a heatmap color value, with red indicating positive selection and blue indicating negative selection for significant sites.

Our first question was “*How are genes and sites evolving across the entire dataset, and is the evolutionary pattern we find consistent with what we already know about IAV*?” We confirm a few expected evolutionary patterns: most genes showed evidence of strong purifying selection, with mean *ω* values in the range 0.0627 to 0.8193 for the foreground (cattle) internal branches and many individual sites evolving under negative selection (Fig. 2, Table S2). However, when we filter for genes with significant BUSTED results, we find that over half of all gene products (PB2, PB1, PA, NA, NS1) also showed some statistical evidence of episodic diversifying selection for at least one site on at least one branch. At the site level, there is evidence that one or more biochemical properties from the set containing secondary structure, bipolar, molecular volume, amino acid composition, and electrostatic charge are under selection for at least one site in each gene.

**Fig. 3.**
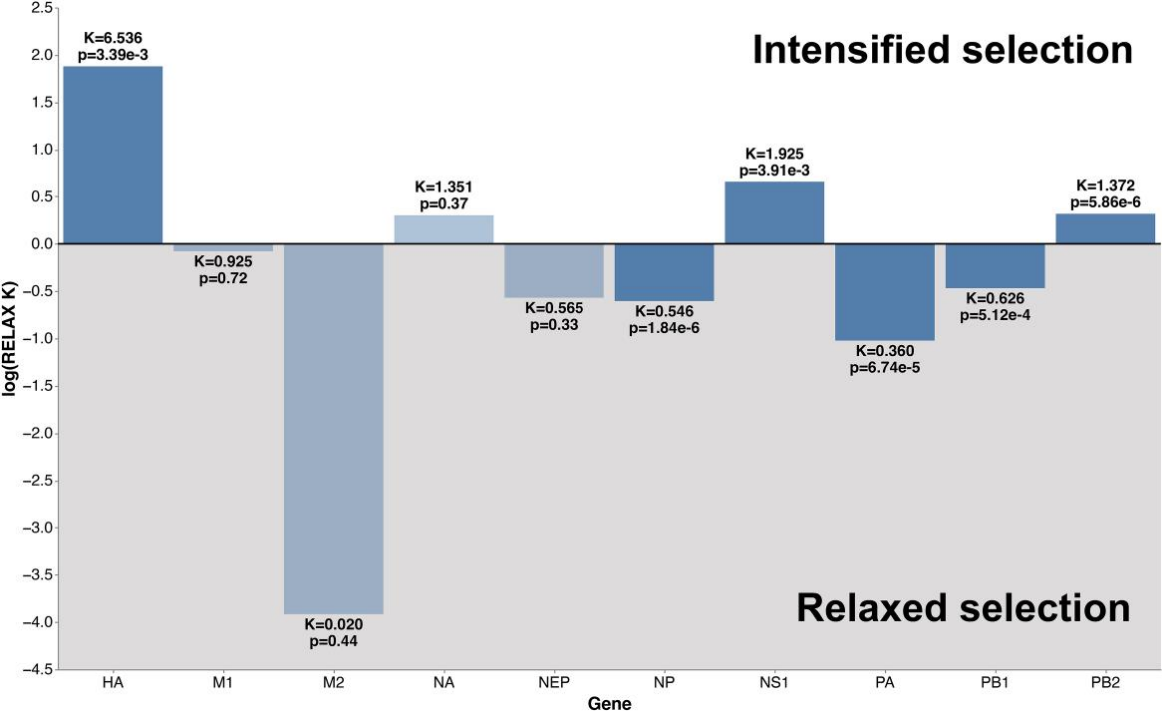
RELAX results for H5N1 sequences. The *K*-value is the Foreground relaxation/intensification parameter from the RELAX analysis, plotted here on a log scale. The *K*-value for the Foreground branches is estimated relative to the Reference branches, where *K* is set to 1 (not shown). Lower *K*-values (log(K)<0) indicate relaxed selection compared to the Reference branches, while higher *K*-values (log(K)>0) indicate intensified—but not *necessarily* positive—selection. p<0.05 indicates that the differences are significant.

Our second question was “*How does IAV evolution differ between the wild bird reservoir hosts and the cattle outbreak*?” RELAX analysis suggests that H5N1 sequences in cattle hosts are under **intensified** selection in HA, NS1, and PB2 and under **relaxed** selection in NP, PA, and PB1, relative to the viral sequences found in wild bird hosts (Fig. 3). Our findings have some precedent: Misra *et al.* independently identified relaxed selection in PB2 internal branches in the entire 2.3.4.4b clade of highly pathogenic avian influenza, relative to other H5N1 clades (Misra et al. 2024). We also filtered our results for individual sites with evidence of intensified positive selection; ie sites with significant MEME test results for episodic positive selection and significant Contrast-FEL test results with $\beta_{\mathrm{cattle}}>\beta_{\mathrm{wild}\;\mathrm{birds}}$. We identified two sites meeting both criteria: in PA (site 655; Table S3) and in M2 (site 88; Table S3). Site 88 of the M2 gene also had a different majority residue among the cattle branches (asparagine) and the wild bird branches (aspartate), possibly indicating a functional change in the protein product. Although neither site meets a conservative 5% FDR threshold, an FDR of 20% is widely considered acceptable in exploratory genomic screens to identify potential adaptive candidates. The M2 gene encodes matrix protein 2, a component of the viral envelope involved in viral genome packaging and the production of infectious viral particles (Hughey et al. 1995; Chen et al. 2008; Ozawa et al. 2009). M2 residues 82 to 89 are specifically required to produce larger titers of infectious virus (McCown and Pekosz 2006), and residues in this region were shown to be under positive selection in human influenza as well (Furuse et al. 2009). All sequence datasets, alignments, phylogenetic trees, configuration files, and raw HyPhy output files generated for this H5N1 case study are publicly available in our results repository at https://github.com/veg/capheine-h5n1.

## Usage and Implementation

After installing Nextflow (a lightweight, ∼40 MB Java runtime), CAPHEINE can be executed on any POSIX-compliant platform. The dependencies of the pipeline (including cawlign, IQ-TREE, and HyPhy) are fully containerized, requiring approximately 600 MB of disk space under Docker, Singularity, or Conda. While this represents a modest local storage footprint, it isolates users from the fragile, manual configuration of individual packages and guarantees bit-wise reproducibility across environments. Additionally, for users who prefer a graphical user interface or want a zero-install deployment, CAPHEINE is also available to run on the Galaxy platform, with the workflow definition hosted in the WorkflowHub aggregator at https://workflowhub.eu/workflows/2111. The pipeline can be run with default parameters using the following command:

nextflow run veg/CAPHEINE} \

-profile <docker/singularity/conda/custom> \

–reference˙genes <reference˙genes.fasta>

–unaligned˙seqs <unaligned˙seqs.fasta> \

–outdir <OUTDIR>

CAPHEINE requires two primary inputs: (i) a FASTA file containing unaligned pathogen genomes and (ii) a FASTA file of reference coding sequences. Optional arguments allow users to specify foreground lineages via a newline-separated list or regular expression pattern. More detailed usage instructions can be found at https://github.com/veg/CAPHEINE. Output consists of per-gene and per-site CSV files summarizing likelihood ratio statistics, parameter estimates, and *p*-values for each HyPhy method. Output files can be viewed in any spreadsheet software package or visualized at https://observablehq.com/@hyphy/drhip-result-viewer

## Discussion

CAPHEINE streamlines exploratory selection evolutionary analysis by bundling the most commonly used HyPhy tests for selection into a single, coherent workflow. With CAPHEINE, researchers can ask—and rigorously answer—routine questions about gene- and site-specific pathogen evolution that would otherwise require bespoke scripting and repeated data wrangling. The integrated analysis suite also supports comparative questions across host reservoirs or epidemic waves, identifying relaxed or intensified selection across genes and pinpointing specific sites with altered biochemical constraints. In our H5N1 case study, CAPHEINE identified statistically significant evidence of episodic selection in the HA viral surface protein and in multiple RNA polymerase subunits. Our workflow easily identified specific sites with host-associated shifts in selection pressure, including site 655 in PA which met both episodic selection and clade-specific intensification criteria. We also identified site 88 in the M2 gene as undergoing episodic selection (MEME  p=0.018) with an associated host-specific change in majority residue (asparagine in cattle vs. aspartate in wild birds), though its clade-specific intensification was not statistically significant (p=0.177). Such residue shifts, even when not showing differential selection rates, provide key candidates for experimental characterization of host adaptation.

A key strength of CAPHEINE is that it transforms a collection of separate, complex command-line utilities into a unified, standardized pipeline, providing a substantial advance in bioinformatics usability and reproducibility. Rather than proposing new selection detection algorithms, CAPHEINE provides the engineering scaffolding necessary to make advanced evolutionary methods accessible to epidemiologists and researchers without specialized bioinformatics expertise, guaranteeing identical execution from raw sequences to MultiQC reports across diverse compute platforms. Because the pipeline is distributed with Conda, Docker, and Singularity options, it runs reproducibly on laptops, HPC clusters, and containerized cloud environments without manual environment tuning. CAPHEINE benefits from the standard configuration patterns, clear provenance, and community-tested orchestration of Nextflow and nf-core, and the workflow’s modular architecture enables robust process management and scalable parallelism. From the user’s perspective, inputs are minimal: just the sequences to analyze and a set of reference coding genes (which can frequently be found within the NCBI genome datasets), plus an optional foreground list or regular expression for contrastive selection tests.

CAPHEINE is agnostic to pathogenicity and can be applied to nonviral, nonpathogenic microbes so long as coding sequences and suitable references are provided. However, users should be aware that CAPHEINE does not account for paralogs among the reference genes, nonhaploid genomes, or species-specific idiosyncrasies—the handling of these is left to the discretion of the species expert. We recommend exploring population genomic methods for evolutionary testing of nonhaploid species. In addition, CAPHEINE does not currently perform automated recombination screening (eg via GARD) or account for processes like horizontal gene transfer (HGT) or hybridization, which result in phylogenetic discordance across the alignment. These evolutionary processes violate the assumption of a single shared tree and can bias selection estimates, typically by inflating false positive rates in site-level selection tests (Kosakovsky Pond et al. 2006). While HyPhy natively supports partitioned analyses using multiple trees to account for such discordance, automating genetic algorithms for recombination breakpoint detection remains computationally prohibitive for the large-scale datasets CAPHEINE is designed to process. For segmented viruses (such as Influenza A), reassortment is accommodated by analyzing segments independently. However, for recombining pathogens, or when HGT or hybridization is suspected, users must perform recombination screening and partition the alignment prior to selection analysis, or use standalone HyPhy tools to fit multi-tree models.

CAPHEINE standardizes pathogen sequence preprocessing and analysis via a comprehensive selection toolkit. Researchers and surveillance teams can analyze new isolates quickly to gain insight about selective pressures, functional hotspots, and lineage-specific shifts. CAPHEINE grants researchers a window into viral evolutionary dynamics, providing actionable leads for experimental follow-up, vaccine and drug target prioritization, and public health decision-making.

## Supplementary Material

evag173_Supplementary_Data

## Data Availability

CAPHEINE can be downloaded from https://github.com/veg/CAPHEINE and installed on any Mac, Linux, or Windows system with Nextflow installed. The workflow is also available to run on the Galaxy platform, with the workflow definition hosted in the WorkflowHub aggregator at https://workflowhub.eu/workflows/2111. Our results repository for the H5N1 case study is available at https://github.com/veg/capheine-h5n1, and contains all sequence datasets, alignments, phylogenetic trees, configuration files, and raw HyPhy output files generated for the case study.

## References

[evag173-B1] Anon . Veg/cawlign: codon-aware alignment. https://github.com/veg/cawlign. 2025.

[evag173-B2] Atchley WR, Zhao J, Fernandes AD, Drüke T. Solving the protein sequence metric problem. Proc Natl Acad Sci USA. 2005:102:6395–6400. 10.1073/pnas.0408677102. 15851683 PMC1088356

[evag173-B3] Balech B et al Investigating the evolutionary dynamics and mutational pattern of SARS-CoV-2 spike gene on selected SARS-CoV-2 variants. PLoS One. 2025:20:e0333093. 10.1371/journal.pone.0333093. 41118444 PMC12539718

[evag173-B4] Burke DS . Recombination in HIV: an important viral evolutionary strategy. Emerg Infect Dis. 1997:3:253–259. 10.3201/eid0303.970301. 9284369 PMC2627633

[evag173-B5] Chen BJ, Leser GP, Jackson D, Lamb RA. The influenza virus M2 protein cytoplasmic tail interacts with the M1 protein and influences virus assembly at the site of virus budding. J Virol. 2008:82:10059–10070. 10.1128/JVI.01184-08. 18701586 PMC2566248

[evag173-B6] da Silva AF et al ViralFlow v1.0—a computational workflow for streamlining viral genomic surveillance. NAR Genom Bioinform. 2024:6:lqae056. 10.1093/nargab/lqae056. 38800829 PMC11127631

[evag173-B7] Di Tommaso P et al Nextflow enables reproducible computational workflows. Nat Biotechnol. 2017:35:316–319. 10.1038/nbt.3820. 28398311

[evag173-B8] Dingens AS, Arenz D, Weight H, Overbaugh J, Bloom JD. An antigenic atlas of HIV-1 escape from broadly neutralizing antibodies distinguishes functional and structural epitopes. Immunity. 2019:50:520–532.e3. 10.1016/j.immuni.2018.12.017. 30709739 PMC6435357

[evag173-B9] Drabeck DH et al Metabolomics-guided genomic comparisons reveal convergent evolution of hibernation genes in mammals. Mol Biol Evol. 2025:42:msaf188. 10.1093/molbev/msaf188. 40812782 PMC12379893

[evag173-B10] Ewels PA et al Nf-core: community curated bioinformatics pipelines. 2019. 10.1038/s41587-020-0439-x 32055031

[evag173-B11] Furuse Y, Suzuki A, Kamigaki T, Oshitani H. Evolution of the M gene of the influenza A virus in different host species: large-scale sequence analysis. Virol J. 2009:6:67. 10.1186/1743-422X-6-67. 19476650 PMC2694789

[evag173-B12] Hadfield J et al Nextstrain: real-time tracking of pathogen evolution. Bioinformatics. 2018:34:4121–4123. 10.1093/bioinformatics/bty407. 29790939 PMC6247931

[evag173-B13] Holmes EC . The comparative genomics of viral emergence. Proc Natl Acad Sci USA. 2010:107:1742–1746. 10.1073/pnas.0906193106. 19858482 PMC2868293

[evag173-B14] Huang S et al Positive selection analyses identify a single WWE domain residue that shapes ZAP into a more potent restriction factor against alphaviruses. PLoS Pathog. 2024:20:e1011836. 10.1371/journal.ppat.1011836. 39207950 PMC11361444

[evag173-B15] Hughey PG et al Effects of antibody to the influenza A virus M2 protein on M2 surface expression and virus assembly. Virology. 1995:212:411–421. 10.1006/viro.1995.1498. 7571410

[evag173-B16] Jetzt AE et al High rate of recombination throughout the human immunodeficiency virus type 1 genome. J Virol. 2000:74:1234–1240. 10.1128/jvi.74.3.1234-1240.2000. 10627533 PMC111457

[evag173-B17] Katz K et al The sequence read archive: a decade more of explosive growth. Nucleic Acids Res. 2022:50:D387–D390. 10.1093/nar/gkab1053. 34850094 PMC8728234

[evag173-B18] Kosakovsky Pond SL, Frost SDW. Not so different after all: a comparison of methods for detecting amino acid sites under selection. Mol Biol Evol. 2005:22:1208–1222. 10.1093/molbev/msi105. 15703242

[evag173-B19] Kosakovsky Pond SL et al Hyphy 2.5—a customizable platform for evolutionary hypothesis testing using phylogenies. Mol Biol Evol. 2020:37:295–299. 10.1093/molbev/msz197. 31504749 PMC8204705

[evag173-B20] Kosakovsky Pond SL, Posada D, Gravenor MB, Woelk CH, Frost SDW. Gard: a genetic algorithm for recombination detection. Bioinformatics. 2006:22:3096–3098. 10.1093/bioinformatics/btl474. 17110367

[evag173-B21] Kosakovsky Pond SL et al Contrast-FEL—a test for differences in selective pressures at individual sites among clades and sets of branches. Mol Biol Evol. 2021:38:1184–1198. 10.1093/molbev/msaa263. 33064823 PMC7947784

[evag173-B22] Kotwa JD et al High host specificity of alphacoronaviruses in Nearctic, insectivorous bats. Npj Viruses. 2025:3:38. 10.1038/s44298-025-00115-y.

[evag173-B23] Langedijk AC et al The genomic evolutionary dynamics and global circulation patterns of respiratory syncytial virus. Nat Commun. 2024:15:3083. 10.1038/s41467-024-47118-6. 38600104 PMC11006891

[evag173-B24] Lee JM et al Mapping person-to-person variation in viral mutations that escape polyclonal serum targeting influenza hemagglutinin. Elife. 2019:8:e49324. 10.7554/eLife.49324. 31452511 PMC6711711

[evag173-B25] Leventhal GE, Hill AL, Nowak MA, Bonhoeffer S. Evolution and emergence of infectious diseases in theoretical and real-world networks. Nat Commun. 2015:6:6101. 10.1038/ncomms7101. 25592476 PMC4335509

[evag173-B26] Lucaci AG et al Rascl: rapid assessment of selection in clades through molecular sequence analysis. PLoS One. 2022:17:e0275623. 10.1371/journal.pone.0275623. 36322581 PMC9629619

[evag173-B27] Luciani F, Alizon S, Fraser C. The evolutionary dynamics of a rapidly mutating virus within and between hosts: the case of hepatitis C virus. PLoS Comput Biol. 2009:5:e1000565. 10.1371/journal.pcbi.1000565. 19911046 PMC2768904

[evag173-B28] Markov PV et al The evolution of SARS-CoV-2. Nat Rev Microbiol. 2023:21:361–379. 10.1038/s41579-023-00878-2. 37020110

[evag173-B29] McCown MF, Pekosz A. Distinct domains of the influenza a virus M2 protein cytoplasmic tail mediate binding to the M1 protein and facilitate infectious virus production. J Virol. 2006:80:8178–8189. 10.1128/JVI.00627-06. 16873274 PMC1563831

[evag173-B30] Minh BQ et al IQ-TREE 2: new models and efficient methods for phylogenetic inference in the genomic era. Mol Biol Evol. 2020:37:1530–1534. 10.1093/molbev/msaa015. 32011700 PMC7182206

[evag173-B31] Misra S, Gilbride E, Ramasamy S, Pond SLK, Kuchipudi SV. Enhanced diversifying selection on polymerase genes in H5N1 clade 2.3.4.4b: a key driver of altered species tropism and host range expansion. 2024.

[evag173-B32] Murrell B et al Gene-wide identification of episodic selection. Mol Biol Evol. 2015:32:1365–1371. 10.1093/molbev/msv035. 25701167 PMC4408417

[evag173-B33] Murrell B et al Detecting individual sites subject to episodic diversifying selection. PLoS Genet. 2012:8:e1002764. 10.1371/journal.pgen.1002764. 22807683 PMC3395634

[evag173-B34] Nanaware N, Banerjee A, Mullick Bagchi S, Bagchi P, Mukherjee A. Dengue virus infection: a tale of viral exploitations and host responses. Viruses. 2021:13:1967. 10.3390/v13101967. 34696397 PMC8541669

[evag173-B35] Ozawa M et al Nucleotide sequence requirements at the 5’ end of the influenza A virus M RNA segment for efficient virus replication. J Virol. 2009:83:3384–3388. 10.1128/JVI.02513-08. 19158245 PMC2655591

[evag173-B36] Policarpo M, Baldwin MW, Casane D, Salzburger W. Diversity and evolution of the vertebrate chemoreceptor gene repertoire. Nat Commun. 2024:15:1421. 10.1038/s41467-024-45500-y. 38360851 PMC10869828

[evag173-B37] Posada-Céspedes S et al V-pipe: a computational pipeline for assessing viral genetic diversity from high-throughput data. Bioinformatics. 2021:37:1673–1680. 10.1093/bioinformatics/btab015. 33471068 PMC8289377

[evag173-B38] Pybus OG, Rambaut A. Evolutionary analysis of the dynamics of viral infectious disease. Nat Rev Genet. 2009:10:540–550. 10.1038/nrg2583. 19564871 PMC7097015

[evag173-B39] Shu Y, McCauley J. GISAID: global initiative on sharing all influenza data—from vision to reality. Euro Surveill. 2017:22:30494. 10.2807/1560-7917.ES.2017.22.13.30494. 28382917 PMC5388101

[evag173-B40] Siozios S et al Genome dynamics across the evolutionary transition to endosymbiosis. Curr Biol. 2024:34:5659–5670.e7. 10.1016/j.cub.2024.10.044. 39549700

[evag173-B41] Spielman SJ et al Evolution of viral genomes: interplay between selection, recombination, and other forces. Springer; 2019. p. 427–468. 10.1007/978-1-4939-9074-0_14 31278673

[evag173-B42] Steel J, Lowen AC. Influenza a virus reassortment. Springer International Publishing; 2014. p. 377–401. 10.1007/82_2014_395 25007845

[evag173-B43] Wertheim JO, Murrell B, Smith MD, Kosakovsky Pond SL, Scheffler K. Relax: detecting relaxed selection in a phylogenetic framework. Mol Biol Evol. 2015:32:820–832. 10.1093/molbev/msu400. 25540451 PMC4327161

[evag173-B44] West J et al Characterization of changes in the hemagglutinin that accompanied the emergence of H3N2/1968 pandemic influenza viruses. PLoS Pathog. 2021:17:e1009566. 10.1371/journal.ppat.1009566. 34555124 PMC8491938

