# Supplementary material for: CAPHEINE, or Everything and the Kitchen Sink: A Workflow for Automating Selection Analyses Using HyPhy
Source: Genome Biol Evol. 2026 Jul 17;18(8):evag173. doi: 10.1093/gbe/evag173 (PMC13427765; doi:10.1093/gbe/evag173)
Supplement: evag173_Supplementary_Data [file evag173_supplementary_data.pdf]

Supplement

Table S1: **Number of H5N1 sequences per gene after removing duplicates and sequences with more than 50% gaps or ambiguous nucleotides.**

Gene	Sequences
**PB2**	4311
**PB1**	4190
**PA**	4284
**NP**	3390
**NA**	4047
**HA**	4594
**M2**	980
**M1**	2076
**NEP**	1430
**NS1**	2574

Table S2: **Overall selection characterization of H5N1 internal branches. sites**: the number of codon sites in the alignment. Sites under positive selection have been inferred using `MEME` ($q \leq 0.2$), negative selection using `FEL` ($q \leq 0.2$), and clade-specific $\omega_{\text{clade}} > \omega_{\text{other clade}}$ using `Contrast-FEL` ($q \leq 0.2$). `BUSTED` **EDS (p / q)**: the raw p-value and the across-gene false discovery rate (FDR) adjusted q-value for episodic diversifying selection (EDS) on internal branches, and the percent of branch sites for which $\omega \geq 1$. **branches**: the host-specific number of non-zero length branches included for selection testing. **Tree length**: the cumulative length of all branches assigned to each host (`contrast-FEL`, scaled in expected substitutions/nucleotide site). ω: mean estimate on host-specific internal branches (MG94xREV model). Significance threshold is set to $q \leq 0.2$ to facilitate exploratory analysis.

Gene	Sites	Sites under selection ($q \leq 0.2$)			BUSTED EDS	Host	Branches	Tree length	ω
		pos.	neg.	clade specific	(p / q)				
**HA**	568	6	466	2	N.S.	**Cattle**	139	0.1012	0.2649
						**Wild birds**	1799	3.7689	0.1356
**M1**	252	1	192	1	N.S.	**Cattle**	46	0.0612	0.0625
						**Wild birds**	635	1.7726	0.0533
**M2**	97	14	20	1	N.S.	**Cattle**	7	0.0138	1.1916
						**Wild birds**	267	1.1929	0.7929
**NA**	469	6	363	1	0.0012 / 0.0023 (2.18%)	**Cattle**	123	0.1141	0.2344
						**Wild birds**	1462	4.2578	0.1862
**NEP**	121	2	58	1	N.S.	**Cattle**	19	0.0569	0.3941
						**Wild birds**	397	2.0617	0.3061
**NP**	498	2	428	1	N.S.	**Cattle**	102	0.0801	0.1506
						**Wild birds**	1210	2.6860	0.0444
**NS1**	230	26	116	0	0.0002 / 0.0006 (12.63%)	**Cattle**	71	0.1098	0.7334
						**Wild birds**	800	3.0148	0.3172
**PA**	716	0	599	2	7.2×10^{-16} / 3.6×10^{-15} (1.20%)	**Cattle**	166	0.1012	0.2048
						**Wild birds**	1591	2.9522	0.0912
**PB1**	757	0	662	1	0.0003 / 0.0007 (0.85%)	**Cattle**	181	0.0944	0.1390
						**Wild birds**	1582	2.9559	0.0631
**PB2**	759	2	673	1	$< 10^{-16}$ / $< 10^{-15}$ (0.82%)	**Cattle**	196	0.1055	0.1837
						**Wild birds**	1605	3.6323	0.0600

Table S3: **Individual sites which show positive selection on the H5N1 cattle host internal branches (`MEME` q-value ≤ 0.2), have significant physicochemical property changes (`PRIME` q-value ≤ 0.2), or where selection is operating differently on H5N1 cattle host internal branches compared to H5N1 wild bird host internal branches (`Contrast-FEL` marker ≤ 0.2). Codon**: codon position in the multiple sequence alignment; **pos.**: `MEME` q-value; **dif.**: `Contrast-FEL` q-value; **composition**: amino-acid composition of cattle and wild bird sequences at this site; **substitutions**: inferred substitutions on cattle and wild bird clade internal branches at this site. *: a site is marked with * if it is **both** positively selected and intensified ($\beta_{\text{cattle}} > \beta_{\text{wild birds}}$) in cattle sequences; #: the majority residue is different between cattle and wild bird sequences. '-' : test result is not significant, or no substitutions occurred within a group.

Gene	Codon	pos.	dif.	`PRIME`	Composition		Substitutions	
					Cattle	Wild birds	Cattle	Wild birds
**HA**	10	0.018	-	-	I/39<br>-/5<br>M/2<br>?/1	I/1992<br>M/30<br>V/14<br>-/136<br>T/440<br>L/2<br>A/232<br>?/2<br>K/1<br>S/3	I:M/1	I:M/5<br>I:T/4<br>I:V/2<br>A:T/1<br>S:T/1<br>M:V/1
	99	-	-	-	A/35<br>I/12	A/2347<br>T/29<br>D/155<br>S/32<br>P/2<br>I/276<br>N/1<br>V/2<br>-/6<br>?/2	-	A:S/1<br>A:D/7<br>D:D/3<br>A:A/5<br>A:I/1<br>I:I/1<br>A:T/5
	105	-	-	overall, bipolar, structure, volume, composition, charge / < 0.001	L/47	L/2833<br>F/1<br>Q/10<br>I/2<br>-/6	-	L:L/4<br>L:Q/3
	144	0.174	-	-	S/35<br>-/12	S/2694<br>-/139<br>N/1<br>T/14<br>?/1<br>F/2<br>P/1	-	S:T/2<br>S:S/1<br>F:S/1
	146	-	-	overall / 0.034	G/47	G/2834<br>-/7<br>P/9<br>R/1<br>W/1	-	G:G/13<br>G:R/1<br>P:R/1

Continued on next page

Table S3—*Continued from previous page*

Codon	pos.	dif.	PRIME	Composition		Substitutions	
				Cattle	Wild birds	Cattle	Wild birds
147	-	0.001	-	V/44<br>M/3	V/2840<br>M/3<br>E/1<br>-/7<br>A/1	-	V:V/4
149	-	-	overall, bipolar, structure, volume, charge / < 0.001	A/35<br>S/12	A/2269<br>S/575<br>-/8	-	A:S/7<br>A:A/6<br>S:S/7
157	-	-	overall, volume, charge / 0.001	P/35<br>S/12	P/1664<br>S/1180<br>-/7<br>?/1	-	P:P/2<br>P:S/9<br>S:S/4
170	-	-	overall / 0.011	N/36<br>D/11	N/2179<br>D/642<br>E/3<br>S/6<br>G/12<br>-/5<br>Q/1<br>?/3<br>H/1	N:N/1	D:N/21<br>N:N/10<br>N:S/2<br>D:G/1
172	-	0.018	-	A/41<br>T/6	A/2421<br>E/1<br>V/3<br>T/400<br>-/5<br>S/18<br>?/1<br>P/1<br>G/1<br>I/1	A:T/2	A:A/7<br>A:V/1<br>A:T/7<br>A:S/2<br>T:T/1
200	-	-	overall, structure, composition / < 0.001	A/47	A/2144<br>E/685<br>G/5<br>-/6<br>T/1<br>D/5<br>?/1<br>K/4<br>V/1	-	A:E/6<br>A:A/6<br>A:D/1<br>E:E/4<br>E:K/1
284	-	-	overall, volume / 0.002	G/35<br>E/12	G/1479<br>R/1<br>E/1360<br>?/6<br>D/3<br>K/3	-	E:G/12<br>G:G/4<br>E:E/5
389	-	-	-	K/47	K/2774<br>?/24<br>R/54	-	K:R/9<br>K:K/4
492	-	-	overall / 0.010	N/36<br>-/3<br>D/8	N/2613<br>-/36<br>D/203	-	N:N/4<br>D:N/11

Continued on next page

Table S3—*Continued from previous page*

	Codon	pos.	dif.	PRIME	Composition		Substitutions	
					Cattle	Wild birds	Cattle	Wild birds
	539	-	-	overall, volume / 0.034	A/33<br>-/3<br>V/11	A/1515<br>V/1290<br>-/46<br>I/1	-	A:V/8<br>A:A/4<br>V:V/3
	540	0.018	-	-	A/43<br>-/3<br>T/1	A/2787<br>-/46<br>S/1<br>C/10<br>V/3<br>T/5	-	A:A/6<br>A:C/1<br>A:T/1
	544	0.018	-	-	A/42<br>-/4<br>V/1	A/2125<br>-/52<br>T/11<br>S/11<br>V/648<br>E/1<br>?/2<br>M/1<br>G/1	-	A:T/3<br>A:S/1<br>A:V/4<br>V:V/2<br>A:A/1
	545	0.018	-	-	L/43<br>-/4	L/2784<br>?/1<br>-/54<br>G/1<br>T/9<br>S/1<br>R/1<br>P/1	-	L:L/3<br>L:T/1
	550	0.018	-	-	A/41<br>-/6	A/2764<br>T/2<br>-/72<br>L/1<br>S/12<br>D/1	-	A:S/1
**M1**	8	0.019	-	-	E/103<br>-/3	E/1249<br>-/15<br>R/3<br>?/1	-	E:R/1
	87#	-	0.051	-	N/20<br>T/86	N/1190<br>S/1<br>T/76<br>A/1	N:T/2<br>T:T/1	N:N/3
	242	-	-	-	K/105<br>N/1	K/1235<br>R/3<br>N/21<br>T/2<br>-/7	K:K/1	K:N/5<br>K:K/1<br>K:T/1
**M2**	3	0.098	-	-	I/9<br>?/3	I/714<br>T/20	-	I:T/2
	11	0.091	-	-	T/12	T/719<br>I/14<br>S/1	-	I:T/3
	12#	0.150	-	-	K/12	K/284<br>R/450	-	K:R/3

Continued on next page

Table S3—*Continued from previous page*

Codon	pos.	dif.	PRIME	Composition		Substitutions	
				Cattle	Wild birds	Cattle	Wild birds
13	0.041	-	-	N/12	N/677<br>T/25<br>K/22<br>D/2<br>S/6<br>H/2	-	N:T/4<br>K:N/3<br>H:N/1<br>N:S/1
14#	0.091	-	-	G/12	G/347<br>E/387	-	E:G/7
17	0.150	-	-	C/12	C/717<br>Y/15<br>G/1<br>?/1	-	C:Y/4
20	0.091	-	-	S/12	S/706<br>R/4<br>N/19<br>I/4<br>G/1	-	R:S/1<br>N:S/4<br>I:S/1
21	0.142	-	-	D/12	D/692<br>G/42	-	D:G/5
66	0.191	-	-	E/12	E/633<br>G/13<br>-/2<br>K/6<br>A/78<br>T/2	-	E:G/4<br>E:E/1<br>E:K/2<br>A:E/1
82	0.014	-	-	S/12	S/561<br>N/163<br>C/3<br>I/1<br>-/3<br>?/1<br>D/2	-	N:S/9<br>C:S/1<br>D:S/1
88*#	0.150	0.177	-	N/11<br>-/1	D/662<br>N/26<br>-/25<br>Y/7<br>?/1<br>V/1<br>G/9<br>K/1<br>E/1<br>M/1	D:N/2	D:N/4<br>D:G/1<br>D:Y/2
89	0.098	-	-	G/8<br>-/3<br>C/1	G/643<br>-/27<br>S/43<br>D/13<br>V/6<br>C/1<br>A/1	C:G/1	G:G/2<br>G:V/1<br>G:S/6<br>D:G/3<br>C:G/1

Continued on next page

Table S3—*Continued from previous page*

	Codon	pos.	dif.	PRIME	Composition		Substitutions	
					Cattle	Wild birds	Cattle	Wild birds
	95	0.191	-	-	E/8<br>-/4	E/665<br>-/61<br>P/1<br>V/1<br>K/2<br>G/2<br>Q/1<br>N/1	-	E:E/9<br>E:N/1
	96	0.014	-	overall, bipolar, structure, volume, composition, charge / 0.007	L/8<br>-/4	L/664<br>-/68<br>K/1<br>E/1	-	K:L/1<br>L:L/5<br>E:L/1
**NA**	16	0.160	-	-	V/126<br>I/1<br>-/1<br>A/2<br>G/2	V/2287<br>A/82<br>?/2<br>-/26<br>I/104<br>M/1<br>T/17<br>G/2	G:V/1	A:V/15<br>V:V/6<br>I:V/7<br>I:T/1<br>G:V/1
	49	0.015	-	-	C/112<br>-/20	C/1681<br>-/836<br>N/1<br>T/1<br>F/1<br>Y/1	C:S/1	C:S/1<br>N:S/1<br>N:T/1<br>S:T/1<br>C:C/1
	68	0.005	-	-	N/110<br>-/20<br>E/1<br>D/1	N/1554<br>S/195<br>H/3<br>K/2<br>-/764<br>R/2<br>T/1	E:S/1	N:S/2<br>H:N/1<br>Q:R/1<br>N:T/1<br>S:T/1
	74	0.188	-	-	L/8<br>F/124	F/2237<br>-/10<br>L/153<br>V/4<br>Y/2<br>S/19<br>C/60<br>P/36	-	V:V/1<br>F:S/3<br>F:F/2<br>C:F/2<br>F:P/2<br>F:L/1
	84	0.021	-	-	A/19<br>T/113	T/2224<br>I/17<br>R/1<br>A/160<br>Y/2<br>K/106<br>-/7<br>M/1<br>V/1<br>P/2	A:T/1	I:T/4<br>A:T/6<br>K:T/1<br>T:T/2<br>P:T/1

Continued on next page

Table S3—*Continued from previous page*

	Codon	pos.	dif.	PRIME	Composition		Substitutions	
					Cattle	Wild birds	Cattle	Wild birds
	266	-	-	-	S/132	S/2518<br>T/1<br>Q/2	-	S:S/4
	369	-	0.031	-	S/116<br>-/2<br>I/4<br>V/1<br>N/5<br>R/4	S/2439<br>I/25<br>N/40<br>R/12<br>-/4<br>G/1	I:S/1<br>N:S/1<br>R:S/1	I:S/2<br>S:S/11<br>N:S/2<br>R:S/3
	468	< 0.001	-	overall /<br>0.004	D/128<br>-/3<br>N/1	D/2398<br>-/106<br>T/7<br>?/7<br>G/1<br>N/1<br>A/1	-	D:T/1
**NEP**	7#	0.140	-	-	-	L/1006<br>?/1<br>P/58<br>-/1<br>I/3<br>T/1<br>F/2<br>H/1	-	L:P/5
	14#	0.019	-	-	-	M/727<br>V/239<br>L/2<br>T/5<br>I/6<br>A/40<br>G/29<br>E/1<br>K/2<br>Q/21<br>?/1	-	M:V/7<br>I:M/1<br>G:V/2<br>A:V/2<br>A:M/1<br>M:Q/1
	67#	-	0.069	-	-	E/859<br>G/200<br>K/1<br>D/10<br>N/3	-	E:G/6<br>E:E/2<br>D:E/2<br>D:D/1<br>D:G/1<br>G:G/1
**NP**	129	-	-	-	A/279<br>V/1<br>T/1	A/1964<br>S/38<br>?/7<br>T/1<br>-/11<br>G/1	-	A:A/6<br>A:S/3<br>S:S/1
	245	-	0.054	-	S/277<br>G/4	S/2021<br>G/1	G:S/1	S:S/2
	409	0.015	-	-	Q/281	Q/2001<br>-/15<br>S/6	-	Q:Q/4<br>Q:S/1

Continued on next page

	Codon	pos.	dif.	PRIME	Composition		Substitutions	
					Cattle	Wild birds	Cattle	Wild birds
	495	0.054	-	-	E/278<br>-/3	E/1895<br>D/1<br>-/112<br>?/9<br>R/5	-	E:E/7<br>E:R/1
**NS1**	8	0.193	-	-	S/144<br>-/4	S/1740<br>-/26<br>D/2	-	D:S/1<br>S:S/4
	20	0.147	-	-	K/147<br>-/1	K/1757<br>-/5<br>N/1<br>G/2<br>?/2<br>E/1	-	G:K/1<br>K:K/3
	23	0.138	-	-	A/147<br>-/1	A/1736<br>V/2<br>-/5<br>G/1<br>S/24	-	A:S/1<br>S:S/1
	24	0.050	-	-	D/147<br>-/1	D/1737<br>-/5<br>N/2<br>M/24	-	D:M/1
	27	-	-	overall /<br>0.005	L/144<br>M/3<br>-/1	L/1678<br>M/83<br>Q/2<br>-/5	L:M/1	L:M/9<br>L:L/4
	48	0.005	-	-	S/148	S/1248<br>I/13<br>T/1<br>N/502<br>-/3<br>G/1	-	I:S/2<br>N:S/8
	82	0.022	-	-	A/144<br>-/4	A/1093<br>V/3<br>T/126<br>G/3<br>-/525<br>L/11<br>S/1<br>D/5<br>N/1	-	A:G/1<br>A:T/5<br>A:P/1<br>L:P/2<br>A:A/5<br>A:D/1
	83	0.010	-	-	P/5<br>S/138<br>-/4<br>F/1	S/761<br>P/467<br>L/2<br>-/525<br>K/7<br>Q/4<br>A/1<br>Y/1	-	P:S/2<br>S:T/1<br>K:T/1<br>K:Q/1<br>Q:Q/1<br>S:S/2

Continued on next page

Table S3—*Continued from previous page*

Codon	pos.	dif.	PRIME	Composition		Substitutions	
				Cattle	Wild birds	Cattle	Wild birds
91	0.148	-	-	T/138<br>S/3<br>?/7	T/1754<br>I/3<br>?/5<br>N/1<br>S/2<br>A/3	S:T/1	T:T/5<br>S:T/1<br>A:T/1
127	-	-	overall, structure, composition / 0.002	N/148	N/1163<br>S/8<br>T/524<br>I/8<br>A/21<br>P/1<br>V/5<br>D/12<br>R/21<br>K/4<br>-/1	-	N:S/1<br>N:T/11<br>N:N/3<br>I:T/1<br>S:T/1<br>A:T/2<br>T:T/1<br>T:V/1<br>D:N/1<br>N:R/1<br>K:R/1<br>R:R/1
161	0.177	-	-	S/147<br>L/1	S/1696<br>L/13<br>T/58<br>P/1	-	S:T/3<br>L:S/1<br>S:S/1
166	0.195	-	-	L/148	L/1579<br>I/12<br>F/157<br>M/19<br>V/1	-	I:L/1<br>F:L/3<br>L:M/1<br>I:M/1
189	0.193	-	-	D/145<br>G/1<br>N/2	D/1749<br>N/6<br>G/11<br>Y/1<br>?/1	D:N/1	D:N/1<br>D:G/3<br>D:D/1
197	0.005	-	-	T/147<br>N/1	T/1615<br>I/101<br>A/10<br>N/39<br>V/2<br>?/1	-	I:T/6<br>I:V/1<br>A:T/2<br>N:T/1
202	0.138	-	-	A/145<br>T/1<br>-/2	A/1673<br>T/90<br>V/1<br>S/2<br>-/2	-	A:T/5
207	0.195	-	-	N/146<br>?/2	N/1085<br>H/1<br>?/3<br>D/669<br>G/10	-	D:N/5<br>D:G/1
209	0.005	-	-	D/141<br>G/3<br>N/2<br>?/2	D/1211<br>N/129<br>G/374<br>S/17<br>-/1<br>V/34<br>?/2	D:G/1	D:N/13<br>D:G/10<br>G:S/3<br>D:V/1<br>D:D/2

Continued on next page

Table S3—*Continued from previous page*

Codon	pos.	dif.	PRIME	Composition		Substitutions	
				Cattle	Wild birds	Cattle	Wild birds
210	0.023	-	-	G/148	G/1711<br>W/3<br>E/11<br>R/42<br>-/1	-	G:W/1<br>E:G/2<br>G:R/3
212	0.189	-	-	P/147<br>T/1	P/1093<br>L/500<br>S/7<br>-/1<br>F/2<br>T/163<br>A/1<br>H/1	-	L:P/4<br>P:T/1
213	0.002	-	-	P/144<br>S/4	P/1717<br>S/33<br>T/2<br>L/15<br>-/1	P:S/1	P:S/8<br>L:P/3
214	0.002	-	-	L/148	L/1708<br>F/48<br>H/10<br>-/1<br>Q/1	-	F:L/8<br>H:L/1
215	0.005	-	-	P/147<br>S/1	P/1691<br>H/2<br>L/43<br>S/14<br>-/1<br>F/3<br>T/14	-	L:P/2<br>P:S/2<br>F:L/1<br>P:T/1
216	0.066	-	-	P/147<br>S/1	P/1719<br>Q/2<br>S/44<br>-/1<br>?/2	-	P:S/7
218	0.005	-	-	Q/148	Q/1731<br>-/8<br>K/3<br>?/1<br>R/1<br>W/23<br>P/1	-	Q:W/2
219	0.147	-	-	K/148	K/1647<br>E/41<br>-/3<br>N/77	-	E:K/3<br>K:N/2
226	0.015	-	-	I/147<br>T/1	I/1693<br>?/1<br>T/22<br>V/49<br>-/2<br>S/1	-	I:T/4<br>I:V/5

Continued on next page

Table S3—*Continued from previous page*

	Codon	pos.	dif.	PRIME	Composition		Substitutions	
					Cattle	Wild birds	Cattle	Wild birds
	227	0.138	-	-	E/147<br>G/1	E/1725<br>K/8<br>G/31<br>-/2<br>?/1<br>V/1	-	E:K/1<br>E:G/3
	228	0.005	-	overall, volume / < 0.001	S/145<br>P/3	Y/3<br>S/1704<br>P/59<br>-/2	P:S/1	S:Y/1<br>P:S/9
**PA**	29	-	0.013	-	K/56	K/2143<br>R/3<br>-/15<br>G/1<br>E/1<br>?/1	-	K:K/1
	544	-	-	overall, bipolar, structure, volume, composition, charge / 0.001	E/56	E/2136<br>K/1<br>?/21<br>Q/1<br>R/5	-	E:E/4<br>E:Q/1<br>E:R/1
	655	-	0	-	L/34<br>F/19<br>S/3	L/2141<br>-/18<br>?/1<br>I/2<br>F/2	L:L/1<br>F:S/1	L:L/5<br>F:L/1
**PB1**	64	-	0.108	-	P/520<br>-/1<br>L/6<br>S/3	P/2518<br>?/1<br>-/12<br>L/7<br>S/1<br>H/1	L:P/1<br>P:P/3<br>P:S/1	P:P/4
	215	-	-	overall, bipolar / < 0.001	R/446<br>K/83<br>?/1	R/1921<br>K/607<br>-/9<br>?/1<br>G/1<br>M/1	K:R/2<br>R:R/1	K:R/20<br>R:R/2
**PB2**	2	-	0.075	-	E/317<br>-/16<br>?/2	E/2396<br>?/2<br>-/97<br>G/1<br>D/1<br>S/1<br>T/1	-	E:E/9<br>E:T/1
	463	0.077	-	-	I/333<br>V/1<br>?/1	I/2375<br>V/102<br>M/18<br>L/4	-	I:V/2<br>I:M/4

Continued on next page

Table S3—*Continued from previous page*

Codon	pos.	dif.	PRIME	Composition		Substitutions	
				**Cattle**	**Wild birds**	**Cattle**	**Wild birds**
464	-	-	overall, structure, composition / 0.036	L/334<br>?/1	L/2384<br>?/2<br>M/112<br>S/1	L:L/1	L:L/10<br>L:M/2
759	0.077	-	-	N/329<br>?/1<br>-/5	N/2385<br>-/103<br>D/2<br>Y/2<br>?/5<br>I/2	-	D:N/1
